# Factors Influencing Aqueous Proinflammatory Cytokines and Growth Factors in Uveitic Glaucoma

**DOI:** 10.1371/journal.pone.0147080

**Published:** 2016-01-15

**Authors:** Saori Ohira, Toshihiro Inoue, Keiichiro Iwao, Eri Takahashi, Hidenobu Tanihara

**Affiliations:** Department of Ophthalmology, Faculty of Life Sciences, Kumamoto University, Kumamoto, Japan; Casey Eye Institute, UNITED STATES

## Abstract

**Purpose:**

To analyze the effects of factors on aqueous humor proinflammatory cytokine and growth factor levels in patients with uveitic glaucoma (UG).

**Methods:**

In this cross-sectional study, we enrolled 143 participants: 1) UG patients (*n* = 39); 2) primary open-angle glaucoma (POAG) patients (*n* = 36); and 3) cataract surgery patients, as a comparative group (*n* = 68). Aqueous humor samples were obtained at the start of surgery. Aqueous cytokine levels were determined using a multiplex immunoassay (xMAP and the Human Cytokine/Chemokine Panel I).

**Results:**

In UG cases, mean interleukin (IL)-6, IL-8, monocyte chemotactic protein (MCP)-1, tumor necrosis factor (TNF)-α, platelet-derived growth factor (PDGF)-AA, PDGF-AB/BB, and VEGF levels were 171.1, 214.5, 2791.7, 3.5, 23.9, 5.4, and 168.9 pg/mL, respectively, and were higher than those in cataract (non-glaucomatous) cases except PDGF. Levels of IL-6, MCP-1, and VEGF were higher in UG cases than in POAG cases. UG cases with a history of phacoemulsification displayed significantly higher levels of IL-6 (P = 0.0164), IL-8 (P = 0.0003), MCP-1 (P = 0.0465), and PDGF-AB/BB (P = 0.0062) compared to the phakic cases. The presence of cells in the anterior chamber was related to higher levels of IL-8 (P = 0.0002), TNF-α (P = 0.0037), and PDGF-AB/BB (P = 0.0009). The level of PDGF-AB/BB was higher in infectious uveitis than in non-infectious uveitis (P = 0.0211). The level of transforming growth factor (TGF)-β2 was negatively correlated with the levels of MCP-1 (adjusted R^2^ = 0.28, t = -2.45, P = 0.031) and TNF-α (adjusted R^2^ = 0.27, t = -2.43, P = 0.032).

**Conclusion:**

A history of phacoemulsification, the presence of cells in the anterior chamber, and infectious uveitis were related to aqueous proinflammatory cytokine levels in patients with UG. TGF-β2 might be an anti-inflammatory factor in aqueous humor of UG patients.

## Introduction

Uveitis is triggered and/or enhanced by various ocular pathological issues, such as infection with microorganisms, autoimmune reactions, and injury. In uveitic eyes, impairment in visual function is caused by direct inflammatory reactions against ocular tissues, secondary cataracts, and associated glaucoma, uveitic glaucoma (UG). UG is characterized by elevated intraocular pressure (IOP) caused by the uveitis, and the subsequent loss of retinal ganglion cells.

Corticosteroids and IOP-lowering medications are primary therapeutic modalities against UG. In cases refractory to medications, surgical treatments are used [1]. Although controversial surgical results have been reported [2], UG cases may be relatively resistant to surgical treatment, including filtering surgeries, compared to primary open-angle glaucoma (POAG) [3], [4], [5], possibly because chronic inflammatory reactions associated with uveitis can accelerate wound scaring, resulting in failure of the glaucoma surgery. Consistent with this, the uveitic conjunctiva contains significantly more fibroblasts, lymphocytes, and macrophages than controls [6].

Bioactive molecules, such as cytokines and growth factors, are major mediators of inflammatory reactions in the pathology of uveitis [7]. Various bioactive molecules were reportedly elevated simultaneously in the aqueous humor in eyes with uveitis, using a multiplex bead immunoassay [8], [9], [10], [11], [12], [13], [14], a recent advance in measurements of multiple bioactive molecules from a small amount of samples. In the reports, the levels of various cytokines were higher than the controls. In contrast, the levels of active transforming growth factor (TGF)-β2 and SDF-1 (CXCl12) decreased in idiopathic uveitis aqueous humor with increasing inflammation [8]. Min et al. [15] showed the levels of active TGF-β2 in the aqueous humor of POAG and neovascular glaucoma (NVG) were also elevated, whereas those in the aqueous samples of open-angle UG were within the normal range. Recently, we found that multiple cytokines, including IL-6, IL-8, MCP-1, TNF-α, and platelet-derived growth factor (PDGF)-AA, were elevated in the aqueous humor in NVG [16]. However, to the best of our knowledge, there are no reports on the levels of multiple cytokines/growth factors in the aqueous humor in UG and their related background factors.

In previous reports, we clarified that phacoemulsification is a possible prognostic factor for the surgical success of trabeculectomy in open-angle glaucoma [17], [18], [19], [20], and that prolonged elevated aqueous proinflammatory cytokine levels after phacoemulsification was a possible cause for the surgical failure in open-angle glaucoma [21], [22]. Thus, the estimation of aqueous cytokine levels could be informative in the management of glaucoma patients. Here, we report a simultaneous increase in proinflammatory cytokines in aqueous humor samples obtained from eyes with UG, and analyze the effects of a history of ocular surgery and other background factors. We also investigated correlations between cytokine levels and TGF-β2 levels in the aqueous humor of UG.

## Patient and Methods

### Patients

This cross-sectional study was approved by the Institutional Review Board of Kumamoto University. All of the procedures conformed to the Declaration of Helsinki. Written informed consent was obtained from each participating patient. Patients who had undergone trabeculectomy for POAG or UG and aged 20 years old or older were recruited. Cataract patients without systemic diseases (other than hypertension and hyperlipidemia), ocular diseases other than cataracts, a history of ocular surgeries, or an IOP > 21 mmHg were included as controls. IOP was determined using a non-contact tonometer in cataract cases, and a Goldmann tonometer in glaucoma cases. When both eyes of a patient met the inclusion criteria, only the eye treated first was included in the analysis. In all of the participants, the anterior eye segment was examined by glaucoma specialists using a slit-lamp biomicroscope, and all of the changes were recorded. Through dilated pupils, the optic disc was evaluated with a stereo fundus lens to make the diagnosis of glaucoma. The cataract patients and POAG patients coincided with those in our previous report [16].

### Sample Collection

Preoperative aqueous humor was obtained at the start of the phacoemulsification surgery and/or trabeculectomy before any incisional procedures, as previously described [21]. Briefly, aqueous humor was obtained gently at the start of surgery from the anterior chamber, through limbal paracentesis using a syringe with a 30-gauge needle attached. Approximately 70–100 μL was collected in CryoTubes, registered, and stored at -80°C until processing.

### Multiple Immunoassay Analyses

Concentrations of IL-6, IL-8, MCP-1, TNF-α, PDGF-AA, PDGF-BB, and VEGF in the aqueous humor samples were determined using multiplex bead-based immunoassays, xMAP, and Human Cytokine/Chemokine Panel I (Luminex, Austin, TX), and the concentrations of active TGF-β1, TGF-β2, and TGF-β3 were measured using a Multiple Species Panel (Luminex), as previously described [21]. Briefly, a 25 μL aliquot of aqueous humor sample was transferred to a plate, and some of each aliquot was placed into one of the capture microsphere multiplexes. After incubation at 4°C for 18 h, multiplexed cocktails of biotinylated reporter antibodies were mixed, and then incubated at room temperature for 1 h. Multiplexes were developed using an excess of streptavidin-phycoerythrin solution. The solution was mixed with each multiplex, and was then incubated at room temperature for 30 min. Vacuum filtration was used to reduce the volumes of the multiplexed reactions, and then the volumes were increased by dilution with a matrix buffer. A Luminex 200 instrument (Luminex) was used for the analysis, and data were interpreted using proprietary data analysis software (DNASIS Plex, ver. 2.5; Hitachi Software Engineering, Tokyo, Japan).

### Statistical Analysis

Data were analyzed using the JMP V8 statistical software (SAS Institute, Inc., Cary, NC). The Wilcoxon rank-sum test or Fisher’s exact test for two variables and the Tukey-Kramer ‘honestly significantly different’ test for multiple variables were performed. A P value < 0.05 was taken to indicate statistical significance. Graphs were prepared using GraphPad Prism 6 (GraphPad Software, La Jolla, CA).

## Results

### Patient Characteristics and Ophthalmological Data

Aqueous humor samples were obtained from 143 participants: 1) UG patients (*n* = 39); 2) POAG patients (*n* = 36); and 3) cataract surgery patients (non-glaucoma), the comparison group (*n* = 68). Patient characteristics are presented in Table 1. Mean age and duration of glaucoma therapy of UG group were lower, and mean IOP and the number of glaucoma eye drops was significantly higher than the POAG group. The causes of uveitis in the UG cases were sarcoidosis (*n* = 7), ocular herpetic infection (*n* = 3), Behçet’s disease (*n* = 2), and one case each of Vogt-Koyanagi-Harada disease, ocular toxoplasmosis, human T-lymphotropic virus infection, and HLAB27-associated; the causes in other UG cases were unknown (*n* = 23). Medication with a corticosteroid before surgery and the cytokine concentrations for each patient are summarized in Table 2.

**Table 1:** Patient characteristics.

Characteristic	Cataract	POAG	UG
Patients (*n*)	68	36	39
Male/Female	31/37	26/10	19/20*
Age (years)			
Mean ± SD	78.3±6.7	68.9±10.9	60.1±14.8**
Range	61–90	46–88	29–83
Preoperative IOP (mmHg)			
Mean ± SD	11.9±2.9	26.8±7.9	35.9±9.5**
Range	6.5–21.0	11.0–46.0	17.3–53.0
Glaucoma eye drops			
Number, mean ± SD	0	2.9±0.6	3.3±0.5*
Range	0	1–4	2–4
β-blocker, *n* (%)	0	33 (91.7)	38 (97.4)
Prostaglandin analog, *n* (%)	0	34 (94.4)	38 (97.4)
CAI, *n* (%)	0	33 (91.7)	37 (94.9)
α_2_-stimulator, *n* (%)	0	0	13 (33.3)
Others, *n* (%)	0	5 (13.9)	3 (7.7)
Steroid eye drops			
Betamethasone valerate, *n* (%)	0	0	18 (46.2)
Fluorometholone, *n* (%)	0	0	10 (25.6)
Duration of glaucoma therapy (months)			
Mean ± SD	0	105.8±99.2	48.1±47.6**
Range	0	1.3–399.2	0.9–175.9
History of phacoemulsification, *n* (%)	68 (100)	9 (25.0)	14 (35.9)
History of vitrectomy, *n* (%)	0 (0)	0 (0)	2 (5.1)

CAI, carbonic anhydrase inhibitor; IOP, intraocular pressure; UG, uveitic glaucoma; POAG, primary open-angle glaucoma; SD, standard deviation.

**P < 0.01

*P < 0.05, vs. the POAG group.

**Table 2:** Preoperative medications by corticosteroid and cytokine concentrations.

Patient #	Eye drop	Systemic medication	IL-6 (pg/ml)	IL-8 (pg/ml)	MCP-1 (pg/ml)	TNF-α (pg/ml)	PDGF-AA (pg/ml)	PDGF-AB/BB (pg/ml)	VEGF (pg/ml)
1	Betamethasone	None	79	66	2285	3	13	0	55
2	None	None	19	27	3103	2	21	0	19
3	Betamethasone	None	65	174	2041	2	37	0	96
4	Betamethasone	None	46	90	2825	3	14	0	58
5	Betamethasone	None	20	138	2342	2	15	6	27
6	None	None	1	23	1516	1	18	0	31
7	Betamethasone	Prednisolone 5mg	91	684	4853	5	17	5	158
8	Fluorometholone	None	16	18	2814	1	18	0	31
9	Betamethasone	Prednisolone 5mg	441	60	3929	17	27	9	64
10	Fluorometholone	None	9	79	1476	1	23	0	46
11	Betamethasone	Prednisolone 5mg	673	4508	2839	60	32	129	26
12	Betamethasone	None	51	84	3084	1	22	0	258
13	Betamethasone	None	72	344	3643	2	12	0	55
14	None	None	5	34	1105	2	17	0	32
15	Fluorometholone	None	18	50	1783	1	27	0	39
16	None	None	8	84	1155	1	27	0	111
17	None	Prednisolone 30mg	0	7	739	1	28	0	55
18	Betamethasone	None	8	19	1426	1	25	0	38
19	Betamethasone	None	22	28	980	1	19	0	0
20	Betamethasone	None	3	21	1667	1	26	0	56
21	Betamethasone	None	5	15	934	1	24	0	83
22	Betamethasone	None	125	296	5206	1	20	0	81
23	Fluorometholone	None	3	94	184	1	14	45	61
24	Fluorometholone	None	39	28	2116	1	15	0	568
25	Fluorometholone	None	14	10	1274	1	28	0	15
26	Betamethasone	None	328	91	2933	2	54	1	0
27	Fluorometholone	None	974	77	4540	1	35	0	1272
28	None	None	16	26	2537	1	30	0	311
29	None	Prednisolone 30mg	1	8	739	1	28	0	161
30	None	None	95	27	2755	1	17	0	204
31	Betamethasone	None	735	120	3833	1	29	0	130
32	Fluorometholone	None	2	9	820	1	41	0	18
33	Fluorometholone	None	25	35	2331	1	21	0	73
34	None	None	25	16	1833	0	19	0	304
35	None	None	1274	89	7357	1	20	2	159
36	None	Prednisolone 20mg	81	34	4762	5	30	0	141
37	Betamethasone	None	152	653	9694	8	20	15	1419
38	Fluorometholone	None	1004	108	5456	1	23	0	254
39	Betamethasone	None	128	91	3965	3	24	0	77

IL, interleukin; MCP, monocyte chemotactic protein; PDGF, platelet derived growth factor; TNF, tumor necrosis factor; VEGF, vascular endothelial growth factor.

### Biochemical Data

**Fig 1:**
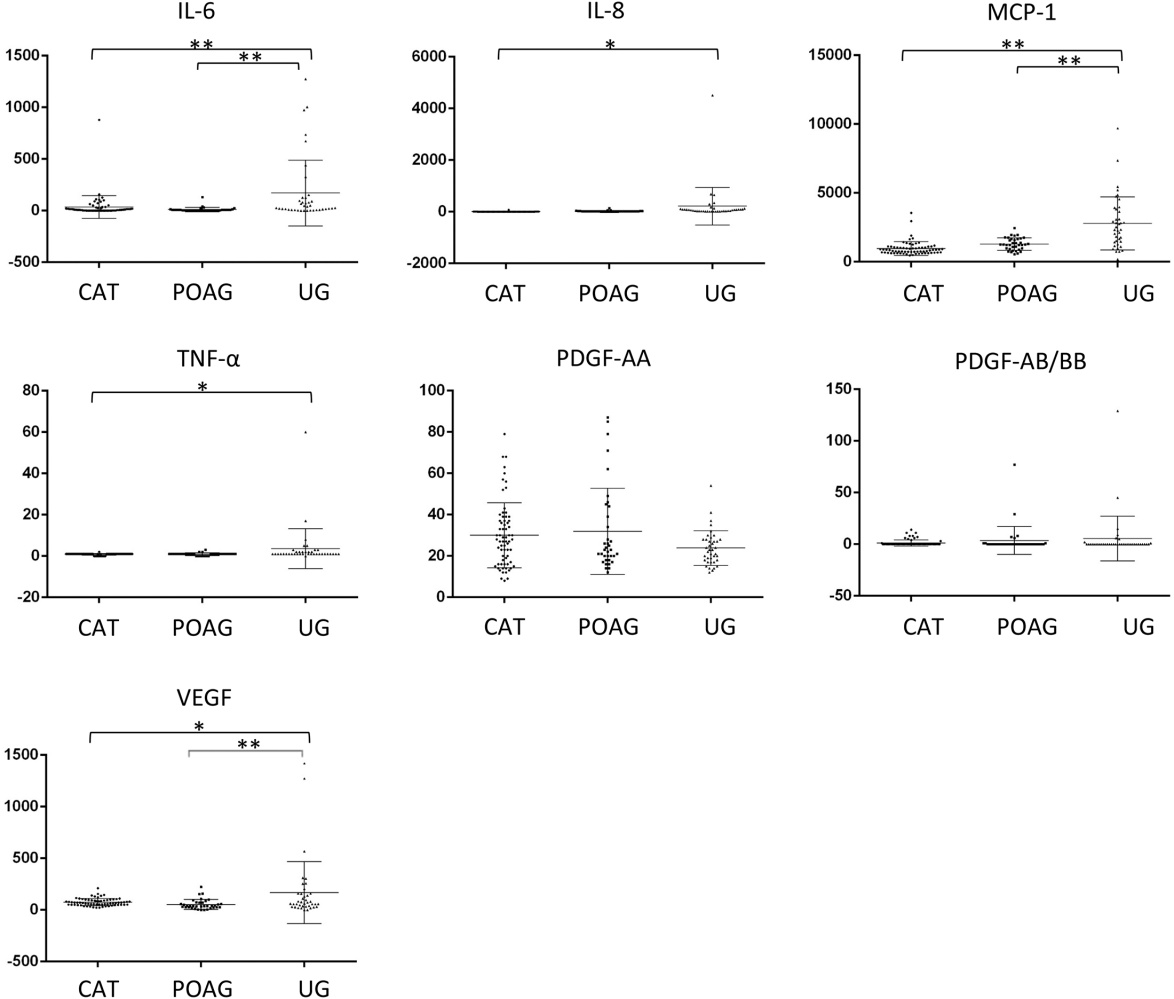
Comparison of levels (pg/mL) of analytes in the eyes of controls (cataractous, CAT) and in eyes with primary open-angle glaucoma (POAG), and uveitic glaucoma (UG). Data points (*n* = 68 for CAT, *n* = 36 for OAG, and *n* = 39 for UG) represent individual samples. Error bars indicate medians ± interquartile range. *P < 0.05. **P < 0.01. IL, interleukin, MCP, monocyte chemotactic protein, PDGF, platelet-derived growth factor, TNF, tumor necrosis factor, VEGF, vascular endothelial growth factor.

In the cataract cases, mean (± SD) IL-6, IL-8, MCP-1, TNF-α, PDGF-AA, PDGF-AB/BB, and VEGF levels were 35.6±109.5, 6.0±9.2, 971.3±500.5, 0.9±0.4, 30.1±15.7, 1.2±3.1, and 75.3±35.0 pg/mL, respectively. In the POAG cases, the corresponding values were 10.0±22.6, 17.0±23.6, 1293.1±445.9, 1.0±0.6, 32.0±20.8, 3.7±13.6, and 52.6±47.7 pg/mL, respectively. In UG cases, the corresponding values were 171.1±317.8, 214.5±721.7, 2791.7±1927.3, 3.5±9.7, 23.9±8.4, 5.4±21.6, and 168.9±298.9 pg/mL, respectively (Fig 1). There were no significant differences in cytokine or growth factor levels between the cataract (non-glaucomatous) and POAG cases. In contrast, the UG cases displayed significantly higher levels of IL-6 (P = 0.0009), IL-8 (P = 0.0179), MCP-1 (P < 0.001), TNF-α (P = 0.0278), and VEGF (P = 0.0112) compared to cataract cases. In addition, the levels of IL-6 (P = 0.0006), MCP-1 (P < 0.001), and VEGF (P = 0.0055) were higher in UG cases than in POAG cases.

**Fig 2:**
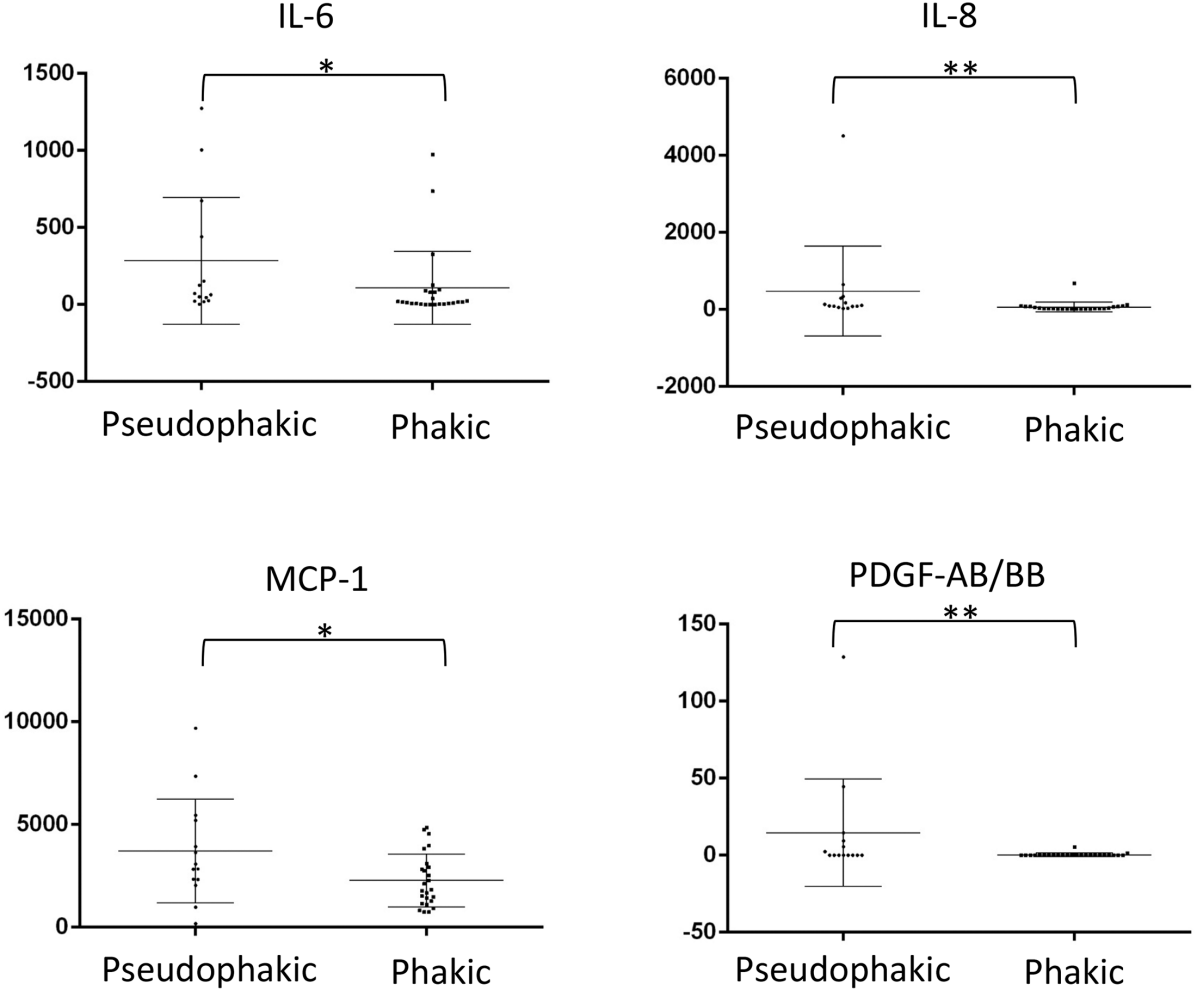
Comparison of levels (pg/mL) of analytes in the eyes of UG cases between with (pseudophakic) and without (phakic) a history of phacoemulsification. Data points (*n* = 14 for with history of phacoemulsification, and 25 for without history of phacoemulsification) represent individual samples. Error bars indicate medians ± interquartile range. *P < 0.05, **P < 0.01. IL, interleukin, MCP, monocyte chemotactic protein, PDGF, platelet-derived growth factor.

**Fig 3:**
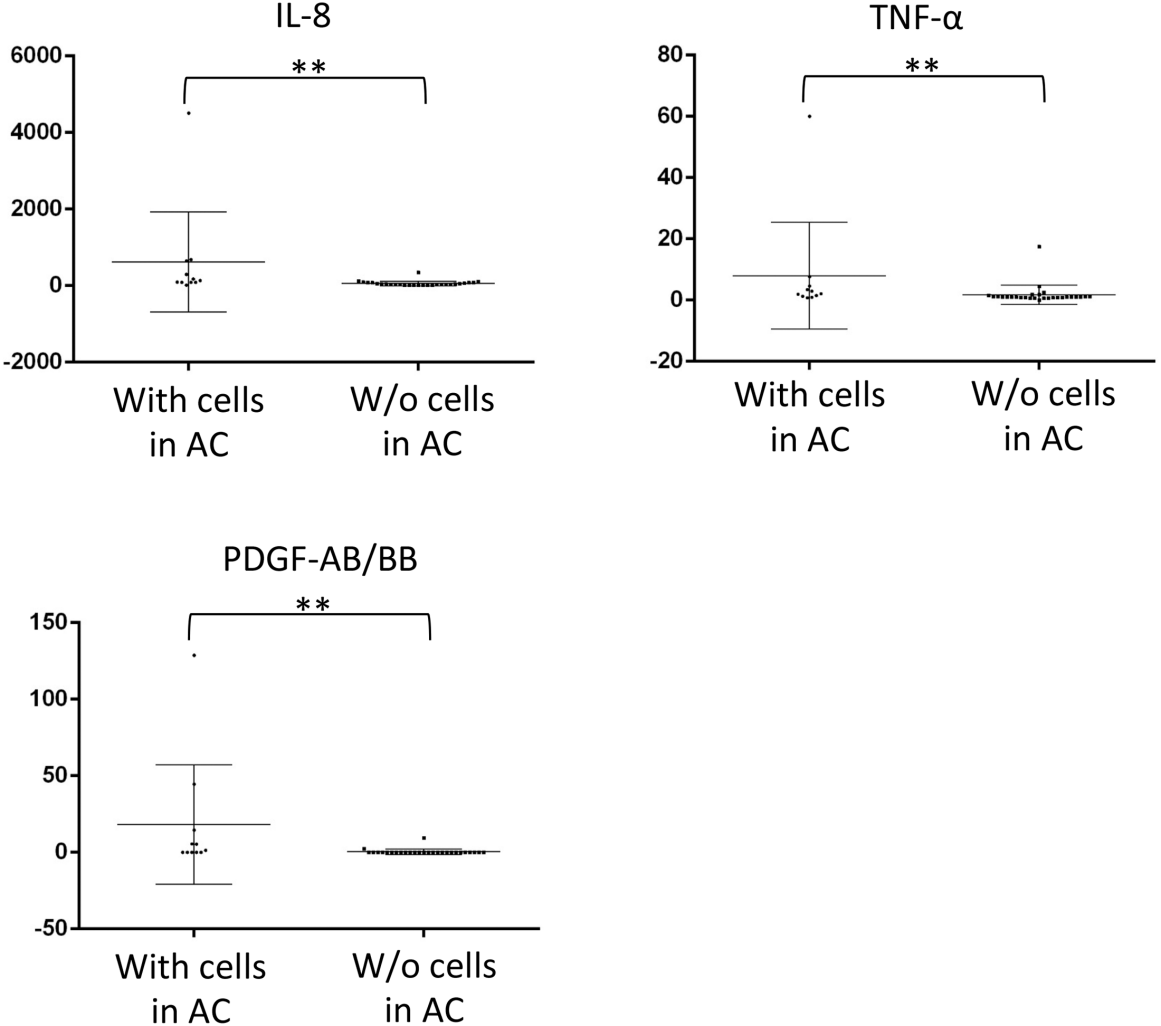
Comparison of levels (pg/mL) of analytes in eyes of UG cases between with and without cells in anterior chamber. Data points (*n* = 11 for with cells and *n* = 28 for without cells in anterior chamber) represent individual samples. Error bars indicate medians ± interquartile range. **P < 0.01. AC, anterior chamber, IL, interleukin, PDGF, platelet-derived growth factor, TNF, tumor necrosis factor.

Compared to UG cases with a history of phacoemulsification (*n* = 14), UG cases without it (*n* = 25) showed significantly higher levels of IL-6 (P = 0.0164), IL-8 (P = 0.0003), MCP-1 (P = 0.0465), and PDGF-AB/BB (P = 0.0062; Fig 2). The mean (± SD) interval between phacoemulsification and subjective trabeculectomy was 53.6±31.9 (range, 6.3–127.5) months. In contrast, there were no significant differences in any cytokine or growth factor level between those with and without a history of vitrectomy. However, UG cases with cells in the anterior chamber (*n* = 11), suggesting the existence of active iritis, showed higher levels of IL-8 (P = 0.0002), TNF-α (P = 0.0037), and PDGF-AB/BB (P = 0.0009; Fig 3).

**Fig 4:**
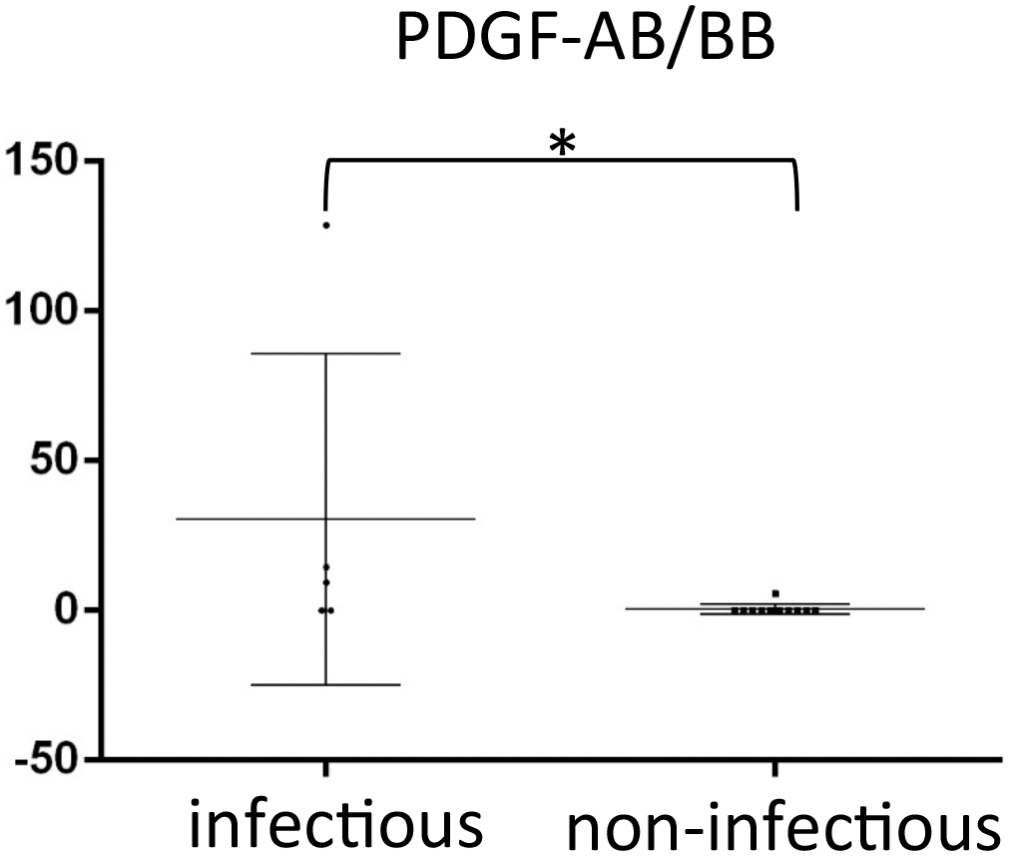
Comparison of levels (pg/mL) of analytes in eyes of UG cases between with infectious and non-infectious uveitis. Data points (*n* = 5 for infectious and *n* = 11 for non-infectious) represent individual samples. Error bars represent medians ± interquartile range. *P < 0.05. PDGF, platelet-derived growth factor.

**Fig 5:**
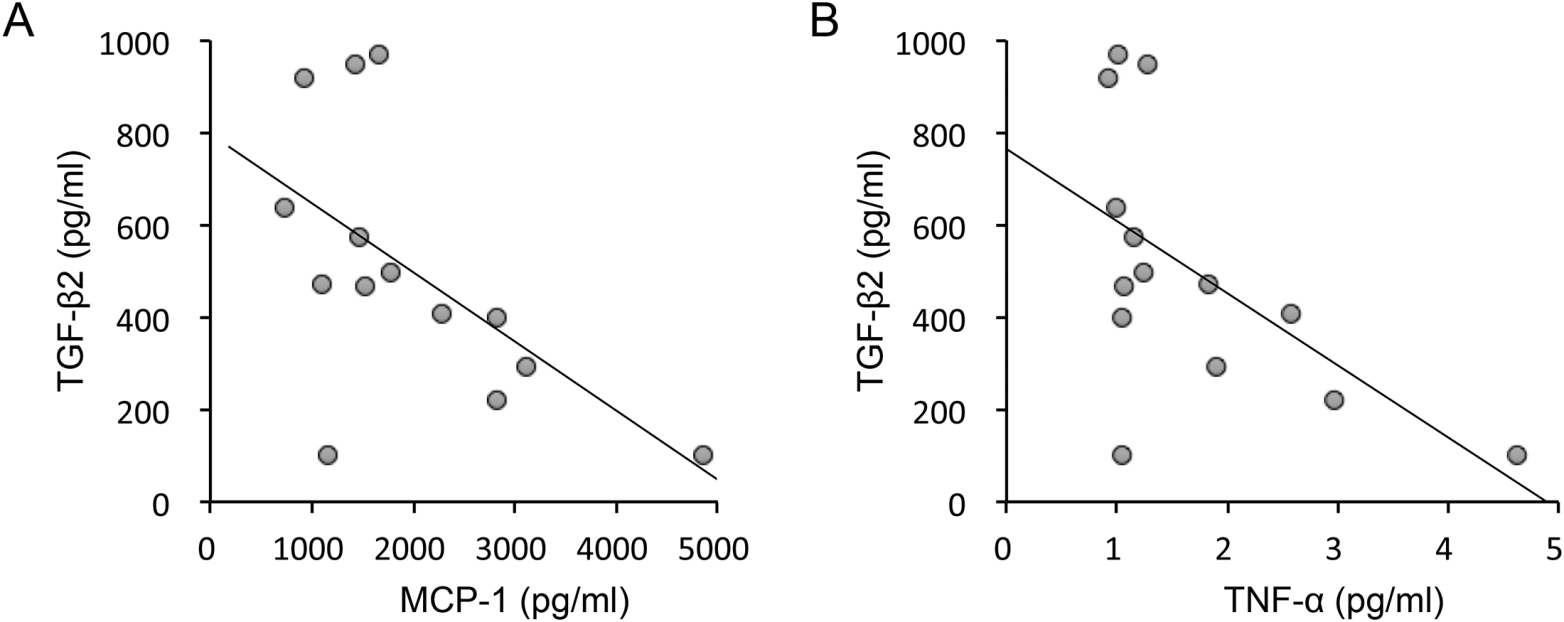
Correlation between the level of TGF-β2 and those of MCP-1 and TNF-α. Scatter plots of the levels of TGF-β2, MCP-1 (A) and TNF-α (B) in aqueous humor of UG (*n* = 14). The level of TGF-β2 negatively correlated with the levels of MCP-1 (adjusted R^2^ = 0.28, t = -2.45, P = 0.031) and TNF-α (adjusted R^2^ = 0.27, t = -2.43, P = 0.032).

UG cases diagnosed as infectious (*n* = 5) showed higher levels of PDGF-AB/BB (P = 0.0211) than UG cases that were diagnosed as non-infectious (*n* = 10; Fig 4), as well as those where the cause of uveitis could not be determined (*n* = 24). However, there were no significant differences in the variables between UG cases with granulomatous uveitis (*n* = 12) and those with non-granulomatous uveitis (*n* = 4). Levels of active TGF-β1, TGF-β2, and TGF-β3 were also measured in some of the UG cases (*n* = 14). The concentration of TGF-β2 was 500.7±277.7 pg/mL, whereas levels of TGF-β1 and -β3 were below the detectable level (12 and 6 pg/mL, respectively) in all of the cases. The level of TGF-β2 was negatively correlated with the levels of MCP-1 (adjusted R^2^ = 0.28, t = -2.45, P = 0.031) and TNF-α (adjusted R^2^ = 0.27, t = -2.43, P = 0.032; Fig 5).

## Discussion

Because cytokines and growth factors are the main mediators of inflammatory reactions, their levels in ocular fluids have been extensively investigated [7]. In the present study, we found that the levels of IL-6, MCP-1, and VEGF in aqueous humor in UG were higher than those in POAG (Fig 1). In our previous study, levels of IL-8 and MCP-1 in the aqueous humor of pseudophakic eyes were higher than in phakic eyes in open-angle glaucoma [21]. In contrast, the levels of cytokines were not related to histories of ocular surgery, including phacoemulsification or vitrectomy in neovascular glaucoma, suggesting that the effect of past intraocular surgery may have been masked by the effect of NVG severity [16]. In the present study, the aqueous levels of IL-6, IL-8, MCP-1, and PDGF-AB/BB were higher in pseudophakic eyes, although the interval between phacoemulsification and trabeculectomy was more than 1 year except in one case (Fig 2), indicating the existence of proinflammatory cytokines in aqueous humor for a prolonged period after cataract surgery in eyes with UG. Because the level of aqueous MCP-1 was related to the surgical success of trabeculectomy in open-angle glaucoma and postoperative modulation of filtering route created by trabeculectomy for aqueous humor [23], [24], the constitutive high levels of inflammatory cytokines in aqueous humor may affect the scaring process of filtering bleb after trabeculectomy in UG similarly. If this is the case, this may partially explain the poor surgical results of trabeculectomy in pseudophakic eyes with UG [3].

Levels of proinflammatory cytokines in the aqueous humor have been reported to correlate with the activity of ocular inflammation in various types of uveitis [25], [26], [27], [28], [29]. Similarly, the levels of IL-8, TNF-α, and PDGF-AB/BB were higher in eyes with aqueous cells, which were detectable by slit-lamp microscopy, versus those without aqueous cells (Fig 3). These results suggest that the presence of aqueous cells can indicate high levels of aqueous cytokines in UG. Because proinflammatory cells such as leukocytes, lymphocytes, and macrophages can produce various cytokines, the cells floating in the aqueous humor may be a source of aqueous cytokines related to the activity of uveitis. An alternative source may be serum, because vascular permeability correlates with inflammatory activity in uveitis [30].

Takase et al. [10] measured the levels of interferon (IFN)-γ, TNF-α, and IL-2, IL-4, IL-5, and IL-10, and found that the expression level of cytokines in aqueous humor was generally higher in infectious uveitis than in non-infectious uveitis. In contrast, in the same study, there was no marked difference in the cytokine expression patterns in the aqueous humor of the different clinical entities of uveitis [10]. These results may not correlate well with the data in the present study; the level of PDGF-AB/BB alone was higher in infectious UG. This may be due to the low diagnostic rate for the cause of uveitis in the present study; it was unknown in 23 of the 39 (59%) cases. Generally, clinicians attempt to suppress the activity of uveitis considerably before glaucoma surgery; thus, the characteristics of each type of uveitis are less prominent at the time point. In addition, a long time period between the diagnosis of uveitis and glaucoma surgery may interfere with the identification of the cause of uveitis in the present study.

In the process of the inflammatory reaction, immunoregulatory cytokines and growth factors, such as IL-10 and interferon-γ, play significant roles in eyes with uveitis. Curnow et al. [8] showed that the levels of both TGF-β2 and CXCL12 in aqueous humor decreased in idiopathic uveitis with increasing inflammation. Furthermore, significantly lower mature TGF-β2 levels were detected in aqueous humor samples of patients with uveitis, compared to the control group without intraocular inflammation [31]. Interestingly, TGF-β suppresses the IL-2-dependent proliferation of T cells by inhibiting the induction of IL-2 and transferrin receptors [32]. In addition, TGF-β suppresses the proliferation of B cells and their secretion of immunoglobulin [33]. Along with neuropeptides such as α-melanocyte-stimulating hormone, vasoactive intestinal peptide, calcitonin gene-related peptide, and somatostatin, TGF-β is thought to contribute partially to the immune-privileged ocular microenvironment [34]. Because TGF-β has immunosuppressive properties, these results suggest that TGF-β2 can suppress the inflammatory activity of uveitis and prevent prolonged inflammatory events. Consistent with this, the level of TGF-β2 correlated negatively with the levels of MCP-1 and TNF-α in aqueous humor in UG in the present study (Fig 5). Another explanation might be that the high levels of inflammatory cytokines reflected the break in the aqueous-vascular barrier, and some proteins that leaked into the aqueous humor from serum counteracted the mature TGF-β. In comparison, TGF-β2 stimulates and activates fibroblasts, resulting in their differentiation into myofibroblasts, and thus contributes to scar formation after a trabeculectomy. Given that TGF-β2 is a multifunctional factor, there is not yet a full understanding of TGF-β2’s roles in the pathophysiology of UG.

Caution should be payed to interpret the data in the present study, because the data analyses in the present study were based on the limited sample number, and the included cases were all Asian patients. Additionally, the low diagnostic yield of the uveitis cases might lead to a biased result as described above. These limitations of the study should be taken into account before generalizing the present data.

In conclusion, a history of phacoemulsification, the presence of cells in the anterior chamber, and infectious uveitis were related to increased aqueous humor proinflammatory cytokine levels in patients with UG. The aqueous TGF-β2 level was negatively correlated with MCP-1 and TNF-α in UG.
